# ‘Creating assessments as an active learning strategy: what are students’ perceptions? A mixed methods study’ – a supplementary letter

**DOI:** 10.1080/10872981.2019.1677392

**Published:** 2019-10-08

**Authors:** Zhen Cahilog, Harry Yeuk Hei Lei, Safa Al-Musawi

**Affiliations:** Imperial College School of Medicine, Imperial College London, London, UK

**Keywords:** Medical education, assessment, multiple-choice questions, student

## Abstract

This is a supplementary letter in response to Kurtz and colleagues’ recent publication exploring the creation of assessments for active learning, with the aim of discussing some of the important points raised by the authors and improvements based on our experience as final year medical students.

Dear Editor,

We have read the recent article by Kurtz and colleagues [1] about student perceptions towards the creation of multiple-choice questions (MCQs) as an active learning strategy with great interest. As final year medical students at Imperial College London, we have found the exercise to be hugely beneficial in improving our understanding of concepts taught in our Pathology course, where teaching is predominantly lecture-based. We have found the creation of these types of questions improved our fact-recall and interpretation of the question stem that would allow us to better narrow down our answer options.

At Imperial College London, medical students are required to complete a 5-day Teaching Skills Course as part of the curriculum. The course covers subject areas such as application of education theory to teaching in different contexts, development and evaluation of teaching plans. As part of the assessment for this course, students choose a challenging topic from the Pathology course and deliver a short fifteen-minute tutorial in groups to peers, followed by MCQs also created by each group to test audience understanding. Consistently, the feedback on this method of learning has been positive and similar to what Kurtz and colleagues found: team-collaboration, coupled with writing MCQs improved our confidence in challenging concepts and expected knowledge, and the majority of our peers enjoyed this in combination with traditional lecture-style teaching as it encouraged deeper learning.

We strongly believe the additional task of teaching a single topic alongside generating focussed MCQs is a great way to consolidate important concepts. Additionally, increasing the specificity of tasks has been previously described as a strategy to reduce the cognitive load required [2], therefore increasing the acceptability to students. Moreover, having peers critique the questions created, with the input of clinical teaching fellows as moderators is hugely beneficial in clarifying the level of detail required for our exams.

One of the common pitfalls of MCQ-based medical examinations is the over-reliance on ‘buzz-words’ which point to a specific diagnosis. In our experience of writing MCQs, we have found this to be crucial for question-makers to bear in mind during exam-writing, as these questions do not test understanding. In view of this, we recommend the use of descriptions of a certain clinical or eponymous sign, along with more comprehensive question stems consisting of parameters used in clinical decision-making, such as basic observations and blood test results. This would be a closer representation of the reality of clinical medicine, which undoubtedly would be more beneficial in preparing medical students for their future career.
